# Concurrent Newborn Hearing and Genetic Screening in a Multi-Ethnic Population in South China

**DOI:** 10.3389/fped.2021.734300

**Published:** 2021-11-30

**Authors:** Xiangrong Tang, Lihua Liu, Sulan Liang, Meie Liang, Tao Liao, Shiqiang Luo, Tizhen Yan, Jianping Chen

**Affiliations:** ^1^Department of Otolaryngology-Head and Neck Surgery, Liuzhou Maternal and Child Health Care Hospital, Liuzhou, China; ^2^Department of Obstetrics, Liuzhou Maternal and Child Health Care Hospital, Liuzhou, China; ^3^Department of Medical Genetics, Liuzhou Maternal and Child Health Care Hospital, Liuzhou, China; ^4^Department of Children's Health Care, Liuzhou Maternal and Child Health Care Hospital, Liuzhou, China

**Keywords:** newborn, hearing loss, hearing screening, limited genetic screening, multi-ethnic population

## Abstract

Hearing loss is a common sensory deficit in humans with intricate genomic landscape and mutational signature. Approximately 1–3 out of 1,000 newborns have hearing loss and up to 60% of these cases have a genetic etiology. In this study, we conducted the concurrent newborn hearing and genetic screening in 20 mutations (18 pathogenic variants in *GJB2, SLC26A4*, and *MT-RNR1* and 2 uncertain clinical significance variants in *GJB3*) for 9,506 normal newborns (4,977 [52.4%] males) from 22 ethnic population in South China. A total of 1,079 (11.4%) newborns failed to pass the initial hearing screening; 160 (1.7%) infants failed to pass the re-screening, and 135 (1.4%) infants presented the diagnostic hearing loss. For the genetic screening, 220 (2.3%) newborns who presented at least one of the screened mutations were more likely to fail the hearing screening and have diagnostic hearing loss than mutation-negative newborns. In comparison to the differences of distribution of mutations, we did not identify any significant difference in the prevalence of screened mutations between Han group (*n* = 5,265) and Zhuang group (*n* = 3,464), despite the lack of number of minority ethnic groups. Studies including larger number of minority ethnic populations are needed in the future.

## Introduction

Hearing loss is a common sensory deficit in humans; it occurs in an estimated 10% of the world's population (500 million individuals) (http://www.who.int/deafness/en/) and affects infants' speech acquisition and cognitive, social, and emotional development (1, 2). Approximately 1–3 out of 1,000 newborns have the hearing loss significant enough to affect speech and language development and up to 60% of these cases have a genetic etiology (3). In addition, hearing loss is one of the most etiologically heterogeneous disorders (4). To date, more than 6,000 mutations in more than 150 genes were proposed to be associated with hearing loss (5).

Early diagnosis and intervention have been shown to be effective in facilitating speech and language development in hearing loss infants and children (6). As a result, the universal newborn hearing screening (UNHS) is now mandated throughout many countries around the world (7–9). However, conventional UNHS is concerned about the missed detection of mild hearing loss, later-onset or drug-induced hearing loss (10); therefore, the targeted genetic pathogenic variants screening should be considered in the UNHS (3, 11).

Nevertheless, it is widely accepted that differential genetic architectures exist between populations (12). Due to the intricate genomic landscape and mutational signature of hearing loss associated genes, it is necessary to consider the ethnic variation when applying targeted variant panels more widely for the screening of newborn hearing loss (13, 14). For instance, although the *GJB2* c.35delG variant is common in Western Europeans, its prevalence is negligible across China and Southeast Asia (15). China has 56 different ethnic groups with apparent genetic differences (the Han group and 55 ethnic minorities) (16, 17); however, few studies reported hearing loss-associated genetic epidemiological differences among various Chinese ethnic groups (18–20).

Guangxi province located in the southwest China has the greatest population of ethnic minorities, including more than 20 groups. Among them, the Zhuang group accounts for about one-third of the total population, which is the largest ethnic minority group in China (http://www.stats.gov.cn/). In this study of concurrent hearing and genetic newborn screening, for the first time, we reported the distributions and characteristics of 20 hearing-loss-related genetic variants in 9,506 newborns from 22 different ethnic groups in Guangxi province.

## Methods

### Study Population and Procedure

**Figure 1:**
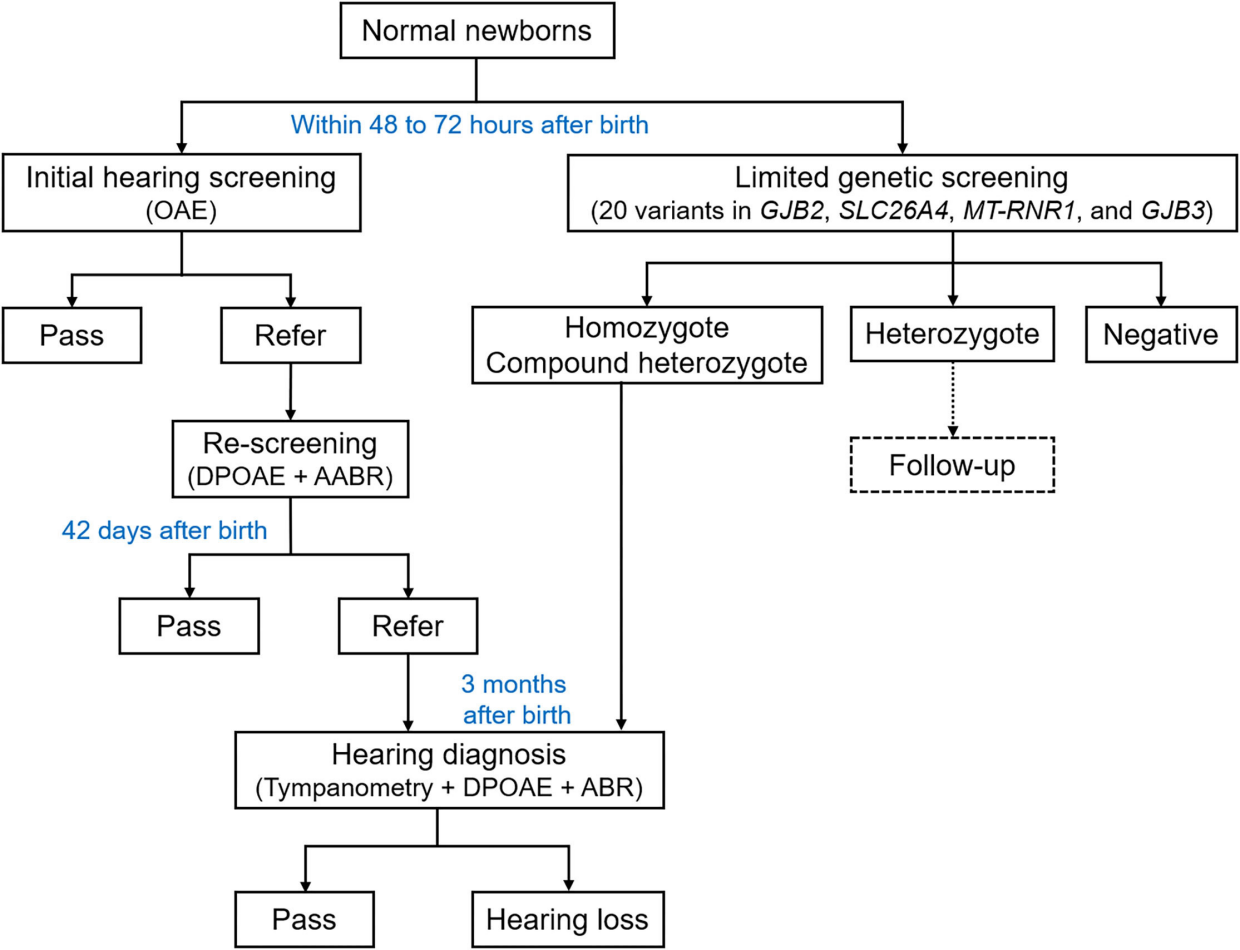
Overall flowchart of the concurrent hearing and genetic study. OAE, otoacoustic emission; DPOAE, distortion product otoacoustic emission; AABR, automated auditory brainstem response; ABR, auditory brainstem response.

This study included a total of 9,506 normal newborns (non-intensive care unit newborns) in Liuzhou Maternal and Child Health Hospital, who received the concurrent hearing and genetic screening from July 2018 to March 2020. The information of ethnicity was collected from the household certificate. Newborn hearing screening and collection of blood spot specimens for genetic screening were conducted after birth as inpatients. Families were notified *via* the clinical report of screening results, and the genetic counseling was offered to those with any genetic mutation. The overall flowchart of screening procedure is shown in Figure 1.

This study was reviewed and approved by the Ethics Committee of Liuzhou Maternal and Child Health Care Hospital (NO. 2018-049). All parents of newborns gave written informed consent in accordance with the Declaration of Helsinki.

### Newborn Hearing Screening

As described previously (9), the newborn initial hearing screening was conducted within 48–72 h after birth. The otoacoustic emission (OAE) test was performed by the trained nurse using the AccuScreen hearing-screening Instrument (Denmark, Otometrics) in a quiet room under background noise lower than 40 dB(A). Newborns who failed to pass the initial screening were referred for re-screening around 42 days after birth using the distortion product otoacoustic emission (DPOAE) and automated auditory brainstem response (AABR) test. Newborns who failed to pass the re-screening were referred to the diagnostic DPOAE and ABR tests conducted by audiological technicians around 3 months after birth. The ABR test was performed using the ICS ChartrEP200 auditory evoked potentials workshop (Denmark, Otometrics).

### Limited Genetic Screening

The genomic DNA was extracted from dried blood spot specimens and genotyped using the Genetic Testing Kit for Hereditary Deafness (China, ZeeSan, 802005) *via* the fluorescent PCR melting curve method. Limited genetic screening included genotyping 20 mutations in *GJB2* (c.35delG, c.167delT, c.176_191del16, c.235delC, and c.299_300delAT), *GJB3* (c.538C>T and c.547G>A), *SLC26A4* (c.2168A>G, c.919-2A>G, c.1174A>T, c.1226G>A, c.1229C>T, c.1707+5G>A, c.1975G>C, c.2027T>A, c.754T>C, c.749T>C, and c.2162C>T), and *MT-RNR1* (m.1494C>T and m.1555A>G). According to the American College of Medical Genetics and Genomics and the Association for Molecular Pathology guidelines, all variants were pathogenic except for two variants in *GJB3*, which were classified as of uncertain clinical significance. All samples that presented any of the 20 screened mutations (positive) were confirmed by Sanger sequencing (provided by ZeeSan Biological Technology Co., Ltd, China). All homozygous and compound heterozygotes were asked to get the hearing diagnosis tests.

### Bioinformatics and Statistical Analysis

Variants were named according to the sequence variant nomenclature recommended by the Human Genome Variation Society. The sequencing data were aligned to the Genome Reference Consortium Human Build 37 (GRCh37/hg19) using the Sequencher software (version 5.1). Statistical analysis was performed by using IBM SPSS version 24.0 software (SPSS Inc., USA). Categorical variables are presented as frequencies and percentages (*n* [%]). The chi-square test was used to compare differences in prevalence of screened mutations among ethnic groups. A two-tailed *p* < 0.05 was considered statistically significant.

## Results

### Overall Hearing and Genetic Screening Results

A total of 9,506 newborns (4,977 [52.4%] males and 4,529 [47.6%] females) underwent the concurrent hearing and genetic screening were included in this study. Among them, a total of 1,079 (11.4%) newborns failed to pass the initial hearing screening, who referred to the re-screening at about 42 days age. Eventually, among 160 (1.7%) infants that failed to pass the re-screening, 128 (1.4%) infants presented the diagnostic hearing loss at about 3 months age. For genetic screening, 220 (2.3%) newborns presented at least one of the 20 screened mutations. To explore the association between abnormal hearing screening outcomes with the presence of mutations, we analyzed the distribution of initial screening, re-screening, and diagnostic hearing loss among mutation positive and negative newborns. In comparison with mutation-negative newborns, those mutation positive newborns showed lower percentage of whom were able to pass the initial hearing screening (*p* = 0.018) and re-screening (*p* = 0.007), and higher prevalence of diagnostic hearing loss (*p* = 0.025), see Table 1.

**Table 1:** Overall results of concurrent hearing and genetic screening.

**Hearing outcomes**	**Mutation negative (*n* = 9,286)**	**Mutation positive (*n* = 220)**	**χ^**2**^**	***p***
Initial screening			5.624	0.018
Pass, *n* (%)	8,243 (88.8)	184 (83.6)		
Refer, *n* (%)	1,043 (11.2)	36 (16.4)		
^a^Re-screening			7.294	0.007
Pass, *n* (%)	894 (85.7)	25 (69.4)		
Refer, *n* (%)	149 (14.3)	11 (30.6)		
Diagnostic hearing loss			4.992	0.025
No, *n* (%)	9,158 (98.6)	213 (96.8)		
Yes, *n* (%)	128 (1.4)	7 (3.2)		

a *A total number of 1,079 newborns failed to pass the initial screening and underwent the re-screening*.

### Characteristics of Different Ethnic Population

Characteristics of the concurrent hearing and genetic screening in 22 different ethnic groups (the Han, Zhuang, Yao, Miao, Dong, Mulao, Hui, Maonan, Shui, Buyi, Tujia, Manchu, Mongolian, Bai, Gelao, Li, Yi, Dai, Jing, She, Naxi, and Korean) from Guangxi province are shown in Table 2. In this study, Han (5,265 [55.4%]) group and Zhuang (3,464 [36.4%]) group comprised the majority of newborns, while the other 20 ethnic groups (777 [8.2%]) account for fewer than a tenth of the total population in this study.

**Table 2:** Results of screening in 21 different ethnic groups.

**Ethnic groups**	**Population number**	**Mutation carrier, *n* (%)**	**Initial screening refer, *n* (%)**	**Re-screening refer, *n* (%)**	**Hearing loss, *n* (%)**
Han	5,265	130 (2.5)	575 (10.9)	75 (1.4)	66 (1.3)
Zhuang	3,464	72 (2.1)	413 (11.9)	68 (2.0)	56 (1.6)
Yao	220	4 (1.8)	19 (8.6)	3 (1.4)	3 (1.4)
Miao	184	3 (1.6)	26 (14.1)	5 (2.7)	4 (2.2)
Dong	183	4 (2.2)	24 (13.1)	6 (3.3)	4 (2.2)
Mulao	108	4 (3.7)	15 (13.9)	1 (0.9)	1 (0.9)
Hui	16	0	0	0	0
Maonan	14	1 (7.1)	1 (7.1)	0	0
Shui	10	0	4 (40.0)	0	0
Buyi	7	0	0	0	0
Tujia	6	1 (16.7)	0	0	0
Manchu	5	0	0	0	0
Mongolian	5	0	1 (20.0)	1 (20.0)	1 (20.0)
Bai	4	0	0	0	0
Gelao	3	1 (33.3)	0	0	0
Li	3	0	1 (33.3)	0	0
Yi	3	0	0	0	0
Dai	2	0	0	0	0
Jing	1	0	0	0	0
She	1	0	0	0	0
Naxi	1	0	0	0	0
Korean	1	0	0	0	0
Total	9,506	220 (2.3)	1,079 (11.4)	160 (1.7)	137 (1.4)

We further analyzed detailed mutant genotypes in each ethnic group, and we did not identify any hearing loss-associated mutant homozygotes. Most of the mutation positive newborns are heterozygotes, and four of whom are compound heterozygotes including two newborns genotyped *SLC26A4* c.1975G>C/c.919-2A>G, one newborn genotyped *SLC26A4* c.919-2A>G/c.754T>C, and one newborn genotyped *MT-RNR1* m.1555A>G/m.1494C>T (Table 3). To understand the hearing loss associated genetic epidemiology of population in this study, we listed the prevalence of those screened mutations in East Asian, South Asian, European (Non-Finnish), Ashkenazi Jewish, and Latino ethnic populations according to the data from the Genome Aggregation Database (gnomAD) (21), and in a Chinese population according to the ChinaMAP (22). The prevalence of most screened mutations in this study is somehow inconsistent with the East Asian and South Asian ethnic populations or total Chinese population (Table 4). In comparison, the prevalence of *SLC26A4* c.1229C>T and c.754T>C seems to be higher, while the prevalence of *GJB3* c.538C>T and c.547G>A seems to be lower in this study.

**Table 3:** Distribution of hearing loss associated mutations in different ethnic groups.

**Mutant genotypes, *n* (%)**	**Han**	**Zhuang**	**Yao**	**Miao**	**Dong**	**Mulao**	**Maonan**	**Tujia**	**Gelao**
	**(*n* = 5,265)**	**(*n* = 3,464)**	**(*n* = 220)**	**(*n* = 184)**	**(*n* = 183)**	**(*n* = 108)**	**(*n* = 14)**	**(*n* = 6)**	**(*n* = 3)**
*GJB2* (NM_004004.5)	61 (1.2)	29 (0.8)	2 (0.9)	2 (1.1)	3 (1.6)	4 (3.7)	0	0	1 (33.3)
c.235de1C/-	46 (0.9)	24 (0.7)	1 (0.4)	2 (1.1)	3 (1.6)	3 (2.8)	0	0	1 (33.3)
c.299_300de1AT/-	13 (0.3)	3 (0.1)	1 (0.4)	0	0	1 (0.9)	0	0	0
c.176_191del16/-	2 (0.04)	2 (0.06)	0	0	0	0	0	0	0
*GJB3* (NM_024009.3)	4 (0.08)	1 (0.03)	0	0	0	0	0	0	0
c.538C>T/-	2 (0.04)	0	0	0	0	0	0	0	0
c.547G>A/-	2 (0.04)	1 (0.03)	0	0	0	0	0	0	0
*SLC26A4* (NM_000441.2)	60 (1.1)	37 (1.1)	2 (0.9)	1 (0.5)	1 (0.6)	0	0	0	0
c.919-2A>G /-	30 (0.6)	16 (0.5)	0	0	1 (0.6)	0	0	0	0
c.1229C>T/-	7 (0.1)	11 (0.3)	1 (0.4)	1 (0.5)	0	0	0	0	0
c.754T>C/-	6 (0.1)	6 (0.2)	1 (0.4)	0	0	0	0	0	0
c.1707+5G>A /-	4 (0.08)	0	0	0	0	0	0	0	0
c.2168 A>G/-	4 (0.08)	0	0	0	0	0	0	0	0
c.1975G>C/-	2 (0.04)	2 (0.06)	0	0	0	0	0	0	0
c.1174 A>T/-	2 (0.04)	1 (0.03)	0	0	0	0	0	0	0
c.1226 G>A/-	1 (0.02)	0	0	0	0	0	0	0	0
c.2162C>T/-	1 (0.02)	0	0	0	0	0	0	0	0
c.749T>C/-	1 (0.02)	0	0	0	0	0	0	0	0
^a^c.1975G>C/c.919-2A>G	2 (0.04)	0	0	0	0	0	0	0	0
^a^c.919-2A>G/c.754T>C	0	1 (0.03)	0	0	0	0	0	0	0
*MT-RNR1* (NC_012920.1)	10 (0.2)	5 (0.1)	0	0	0	0	1 (7.1)	1 (16.7)	0
m.1555A>G/-	7 (0.1)	5 (0.1)	0	0	0	0	1 (7.1)	1 (16.7)	0
m.1494C>T/-	2 (0.04)	0	0	0	0	0	0	0	0
^a^m.1555A>G/m.1494C>T	1 (0.02)	0	0	0	0	0	0	0	0

a *Compound heterozygote*.

**Table 4:** Prevalence of hearing loss-associated mutations in this study, genomAD and ChinaMAP.

**Mutation positive**	**This study**	**gnomAD, %**					**ChinaMAP**
		**East Asian**	**South Asian**	**European (Non-finnish)**	**Ashkenazi Jewish**	**Latino**	
***GJB2*** **(NM_004004.5)**							
c.235de1C	80 (0.8)	0.7	0	0	0	0	0.8
c.299_300de1AT	18 (0.2)	0.1	0	0	0	0	0.1
c.176_191del16	4 (0.04)	0.02	0	0	0	0	No data
***GJB3*** **(NM_024009.3)**							
c.538C>T	2 (0.02)	0.1	0	0	0	0	0.09
c.547G>A	3 (0.03)	0.05	0	0	0	0.4	0.08
***SLC26A4*** **(NM_000441.2)**							
c.919-2A>G	49 (0.5)	0.5	0	0	0	0	0.6
c.1229C>T	20 (0.2)	0.04	0.06	0.01	0.01	0.01	0.05
c.754T>C	14 (0.1)	0.01	0	0	0	0	0.005
c.1707+5G>A	4 (0.04)	0.01	0	0	0	0	0.005
c.2168 A>G	4 (0.04)	0.2	0	0	0	0	0.1
c.1975G>C	5 (0.04)	0.02	0	0	0	0	0.04
c.1174 A>T	3 (0.03)	0.01	0	0	0	0	0.01
c.1226 G>A	1 (0.01)	0.1	0.01	0.01	0	0.02	0.02
c.2162C>T	1 (0.01)	0	0	0	0	0.03	No data
c.749T>C	1 (0.01)	No data	No data	No data	No data	No data	No data
***MT-RNR1*** **(NC_012920.1)**							
m.1555A>G	15 (0.2)	No data	No data	No data	No data	No data	No data
m.1494C>T	3 (0.03)	No data	No data	No data	No data	No data	No data

### Hearing Loss-Associated Mutations in Multiple Ethnic Populations

Although the distribution of hearing loss-associated mutations seemed to be distinct between various ethnic groups (such as mutations on *GJB2*), the disparate number of populations of different ethnic groups might hamper the statistical comparison between majorities with minorities (Table 3). Thus, we further compared the differences of distribution of mutations only between Han group and Zhuang group (Table 5). Although no significant difference in the presence of any of the screened mutations was found between the two ethnic groups, some mutations such as *GJB2* c.299_300de1AT (*p* = 0.087) and *SLC26A4* c.1229C>T (*p* = 0.063) have the tendency of distinguished prevalence between ethnic groups, which needs further study including larger number of subjects in the future.

**Table 5:** Comparison of hearing loss-associated mutations between Han and Zhuang groups.

**Mutation positive, *n* (%)**	**Han (*****n*** **=** **5,265)**		**Zhuang (*****n*** **=** **3,464)**		**χ^**2**^**	***p***
	**Positive**	**Negative**	**Positive**	**Negative**		
Total	130 (2.5)	5,135 (97.5)	72 (2.1)	3,392 (97.9)	1.410	0.235
*GJB2* (NM_004004.5)	61 (1.2)	5,204 (98.8)	29 (0.8)	3,435 (99.2)	2.115	0.146
c.235de1C	46 (0.9)	5,219 (99.1)	24 (0.7)	3,440 (99.3)	0.859	0.354
c.299_300de1AT	13 (0.2)	5,252 (99.8)	3 (0.1)	3,461 (99.9)	2.935	0.087
c.176_191del16	2 (0.04)	5,263 (99.96)	2 (0.06)	3,462 (99.94)	<0.001	0.999
*GJB3* (NM_024009.3)	4 (0.08)	5,261 (99.92)	1 (0.03)	3,463 (99.97)	0.196	0.658
c.538C>T	2 (0.04)	5,263 (99.96)	0	3,464 (100)	0.180	0.671
c.547G>A	2 (0.04)	5,263 (99.96)	1 (0.03)	3,463 (99.97)	<0.001	0.999
*SLC26A4* (NM_000441.2)	60 (1.1)	5,205 (98.9)	37 (1.1)	3,427 (98.9)	0.043	0.836
c.919-2A>G	32 (0.6)	5,233 (99.4)	17 (0.5)	3,447 (99.5)	0.513	0.474
c.1229C>T	7 (0.1)	5,258 (99.9)	11 (0.3)	3,453 (99.7)	3.460	0.063
c.754T>C	6 (0.1)	5,259 (99.9)	7 (0.2)	3,457 (99.8)	1.091	0.296
c.1707+5G>A	4 (0.08)	5,261 (99.92)	0	3,464 (100)	1.235	0.266
c.2168 A>G	4 (0.08)	5,261 (99.92)	0	3,464 (100)	1.235	0.266
c.1975G>C	4 (0.08)	5,261 (99.92)	2 (0.06)	3,462 (99.94)	<0.001	0.999
c.1174 A>T	2 (0.04)	5,263 (99.96)	1 (0.03)	3,463 (99.97)	<0.001	0.999
c.1226 G>A	1 (0.02)	5,264 (99.98)	0	3,464 (100)	<0.001	0.999
c.2162C>T	1 (0.02)	5,264 (99.98)	0	3,464 (100)	<0.001	0.999
c.749T>C	1 (0.02)	5,264 (99.98)	0	3,464 (100)	<0.001	0.999
*MT-RNR1* (NC_012920.1)	10 (0.2)	5,255 (99.8)	5 (0.1)	3,459 (99.9)	0.253	0.615
m.1555A>G	8 (0.1)	5,257 (99.9)	5 (0.1)	3,459 (99.9)	0.008	0.928
m.1494C>T	3 (0.06)	5,262 (99.94)	0	3,464 (100)	0.064	0.415

## Discussion

In this study, we conducted the concurrent newborn hearing and genetic screening in 20 mutations in *GJB2, GJB3, SLC26A4*, and *MT-RNR1* for 9,506 normal newborns from 22 ethnic population in South China.

As a populous country with high incidence of birth defects including hearing loss (23), the national UNHS coverage rate showed an increment from 29.9 to 86.5% between 2008 and 2016 (24) in China. Although the UNHS has played an important role in early detection of congenital hearing loss (7, 10), there are still a proportion of hearing loss population that would be missed by using the conventional UNHS procedure, especially those mild, late-onset hearing loss newborns (3). In the past 10 years, genetic testing has been suggested as one of the most important etiological diagnostic methods for infants and children with hearing loss (25). So far, several large-scale concurrent hearing and genetic newborn screening programs have reported that using the limited genetic screening panel including even a small number of variants in genes commonly associated with hearing loss (*GJB2, GJB3, SLC26A4*, and *MT-RNR1*) could improve the effectiveness of elucidating etiologies, informing high-risk newborns and their maternal relatives of hearing loss (26, 27).

In our results, the total positive rate of limited genetic screened mutations was 2.3%, which was within the comparable range of positive rates in previous studies (18, 28). However, the positive rates of same mutations ranged widely from about 1.5 to 5% (29), indicating the considerable disparity of hearing loss-associated genetic epidemiology in different regions in China. Besides, we observed the potential association between mutant heterozygotes with abnormal hearing screening outcomes, which was always ignored from analysis in previous studies, since the limited genetic screened mutations are regarded as recessive pathogenic or drug susceptible mutations. In addition, the screening panel included only 20 mutations, which could not account for all possible genetic factors associated with hearing loss in this study. It needs further studies to identify if any other risk factors influence the newborn hearing screening outcomes.

It is known that the carrier frequencies of Mendelian disorders, including genetic-associated hearing loss, could be considerably different between various ethnic groups (30). Although there were numerous hearing and genetic screening studies conducted in different regions in China during the past years, few studies focus on the disparity of hearing loss-associated genetic epidemiology in different ethnic groups (20, 28, 31). A recent study indicated the variable population-specific allelic spectra of known hearing loss-associated pathogenic variants using data from 123,136 individuals in seven different ethnic groups (13). To our knowledge, this is the first study that demonstrated the characteristics and distribution of hearing and genetic screening results in a large-scale multiple ethnic population in South China. Although there are no significant differences between the two main ethnic groups in this study, two mutations have the tendency of distinguished distribution in different ethnic groups. In addition, we have reported the prevalence of *SLC26A4* c.2162C>T and c.749T>C in a South Chinese population, which were absent in the ChinaMAP. Since the sample size of some minority ethnic population was too small for comparative analysis, studies including more minority ethnic populations are needed.

In general, these multi-ethnic population characteristics of Guangxi province provide us a good opportunity to explore genomic contributions to hearing loss that vary by different ethnic populations and to promote the application of this knowledge to clinical care in the future (32).

## Conclusions

In this concurrent newborn hearing and genetic screening in a large-scale and multiple ethnic population in South China, we find that newborns who had at least one hearing loss-associated mutation are more likely to fail the hearing screening and have diagnostic hearing loss. However, we did not identify any significant difference in the prevalence of screened mutations between the majority Han ethnic group and Zhuang group, despite the lack of number of minority ethnic groups.

## Data Availability Statement

The original contributions presented in the study are included in the article/supplementary material, further inquiries can be directed to the corresponding author/s.

## Ethics Statement

The studies involving human participants were reviewed and approved by the Ethics Committee of Liuzhou Maternal and Child Health Care Hospital. Written informed consent to participate in this study was provided by the participants' legal guardian/next of kin.

## Author Contributions

XT, TY, and JC contributed to conception and design of the study. LL, SLi, ML, and TL collected the data. XT and SLu performed the genetic and statistical analysis. XT and TY wrote the first draft of the manuscript. JC refined the draft of the manuscript. All authors contributed to manuscript revision, read, and approved the submitted version.

## Funding

This study was supported by grant Z20180014 to XT from the Guangxi Medicine and Health Self-financing Research Project.

## Conflict of Interest

The authors declare that the research was conducted in the absence of any commercial or financial relationships that could be construed as a potential conflict of interest.

## Publisher's Note

All claims expressed in this article are solely those of the authors and do not necessarily represent those of their affiliated organizations, or those of the publisher, the editors and the reviewers. Any product that may be evaluated in this article, or claim that may be made by its manufacturer, is not guaranteed or endorsed by the publisher.
